# Sfrp5 Modulates Both Wnt and BMP Signaling and Regulates Gastrointestinal Organogensis in the Zebrafish, *Danio rerio*

**DOI:** 10.1371/journal.pone.0062470

**Published:** 2013-04-29

**Authors:** Carsten Stuckenholz, Lili Lu, Prakash C. Thakur, Tae-Young Choi, Donghun Shin, Nathan Bahary

**Affiliations:** 1 Department of Medicine, Division of Hematology/Oncology, University of Pittsburgh Medical Center, University of Pittsburgh School of Medicine, Pittsburgh, Pennsylvania, United States of America; 2 Department of Developmental Biology, University of Pittsburgh School of Medicine, Pittsburgh, Pennsylvania, United States of America; Institute of Cellular and Organismic Biology, Taiwan

## Abstract

Sfrp5 belongs to the family of *secreted frizzled related proteins* (Sfrp), secreted inhibitors of Wingless-MMTV Integration Site (Wnt) signaling, which play an important role in cancer and development. We selected *sfrp5* because of its compelling expression profile in the developing endoderm in zebrafish, *Danio rerio*. In this study, overexpression of *sfrp5* in embryos results in defects in both convergent extension (CE) by inhibition of non-canonical Wnt signaling and defects in dorsoventral patterning by inhibition of Tolloid-mediated proteolysis of the BMP inhibitor Chordin. From 25 hours post fertilization (hpf) to 3 days post fertilization (dpf), both overexpression and knockdown of Sfrp5 decrease the size of the endoderm, significantly reducing liver cell number. At 3 dpf, insulin-positive endodermal cells fail to coalesce into a single pancreatic islet. We show that Sfrp5 inhibits both canonical and non-canonical Wnt signaling during embryonic and endodermal development, resulting in endodermal abnormalities.

## Introduction

The Wingless-MMTV Integration Site (Wnt) pathway is a conserved signaling pathway with important roles in development, organogenesis, and carcinogenesis [1]–[5]. Especially in gastrointestinal cancers, upregulation of Wnt signaling is an important early step in tumorigenesis [1], [6], [7]. Wnt proteins are lipid-modified, secreted proteins that bind to Frizzled receptors and activate intracellular signal transduction cascades. One cascade, the canonical signaling pathway, results in stabilization and nuclear localization of β-catenin, frequently causing the activation of pro-proliferative target genes. Another cascade, the non-canonical signaling cascade, results in actin cytoskeletal reorganization and alters the shape and structure of the cell [2], [8].

Given the wide-ranging effects of Wnt signaling, cells regulate it tightly at each step. One evolutionarily conserved family of secreted proteins that modulates Wnt signaling in the extracellular matrix is the family of *secreted frizzled-related proteins* (SFRPs). Sfrp proteins are important for development, such as dorsoventral patterning in zebrafish and *Xenopus laevis* [9]–[12], brain and retina development in zebrafish and medaka [13], [14], gastrulation in amphioxus [15], and formation of mouse epithelial structures and trunk [16]–[18]. They are also frequently dysregulated in cancers [19], [20]. For example, SFRP5 is downregulated by methylation in renal, gastric, and colorectal cancers [21]–[23].

In mammals, SFRP proteins comprise a family of five proteins (SFRP1– SFRP5), which are split into two subfamilies based on sequence homology, one subfamily consisting of SFRP1, 2, and 5 and the other of SFRP3 and 4 [24]. In addition to these, amphibians, such as *Xenopus laevis* and the zebrafish *Danio rerio*, have a third branch of Sfrp proteins, which includes Sizzled and Crescent proteins that play an important role in dorsoventral patterning [11], [12], [25]. Structurally, each SFRP protein consists of two distinct domains, an N-terminal cysteine rich domain (CRD), which is homologous to the extracellular domain of Frizzled proteins, and a second, C-terminal cysteine rich domain with homology to netrin proteins [26]. Both CRD and NTR domains can bind to Wnt signaling molecules [24], [27], [28], with different SFRP proteins binding a different subset of Wnt molecules [29]–[31].

SFRP proteins play complex roles in modulating Wnt signaling [24], [32]. SFRPs can inhibit Wnt signaling [31], [33], but other roles have been demonstrated including potentiation of Wnt signaling by SFRP proteins and biphasic modulation of Wingless signaling by SFRP1 [24], [27]. Binding of SFRP to Wnt proteins can increase the diffusion of Wnt signals in the extracellular space [19], [32]. Finally, SFRP proteins may modulate other secreted signaling molecules. Sfrp3, for example, was shown to bind EGF [34]. In amphibians, the Sfrp family members Sfrp2, Sizzled and Crescent regulate the dorsoventral BMP signaling gradient [9], [11], [12]. Sfrp2 can enhance remodeling of the extracellular matrix [35].

Sfrp5, the focus of this paper, has been shown to bind to the non-canonical Wnt molecules Wnt5a and Wnt11, to inhibit both canonical and non-canonical Wnt signaling in *Xenopus laevis* and in human tissue culture, as well as canonical Wnt signaling in zebrafish [30], [36], [37]. In medaka (*Oryzia latipes*), Sfrp5 is required for normal development of the eye and the tectum, as well as patterning of the optic cup [14]. Sfrp5 transfected into murine fibroblast cells significantly decreased canonical Wnt signaling mediated by Wnt3 [38]. The triple knockout of murine Sfrp1, Sfrp2, and Sfrp5 shows disrupted canonical and non-canonical Wnt signaling, resulting in defects in epithelial development and trunk formation [16], [18].

In our earlier microarray study of gene expression profiles in the developing gastrointestinal (GI) tract of zebrafish, we identified *sfrp5* as an interesting candidate gene because it was highly expressed in endoderm early during GI organogenesis, but its expression decreased with the onset of organ function, suggesting an important role in organogenesis of GI organs [39], [40]. Together with the findings that *SFRP5* is often inactivated in GI cancers and other data underscoring the importance of Wnt signaling in the formation of the zebrafish GI tract [41], these results prompted us to further analyze the role of Sfrp5 in GI organogenesis in zebrafish. In this paper, we report two major findings: First, both increase and knockdown of Sfrp5 result in smaller GI organs, with failure of pancreatic precursor cells to coalesce into a single pancreatic islet in the case of *sfrp5* overexpression. Second, we find that overexpression of *sfrp5* can inhibit BMP signaling by stabilization of the inhibitor Chd and affects dorsoventral patterning.

## Materials and Methods

### Ethics Statement

All studies were carried out in strict accordance with NIH guidelines for animal care and use, and with approval from the University of Pittsburgh Institutional Animal Care and Use Committee (Permits 0902709 and 1202641).

### Zebrafish Husbandry and Injections

1- to 2-cell zebrafish embryos were injected with mRNAs or morpholinos at the indicated concentrations. We used a splice-blocking morpholino targeting the boundary between exon 1 and intron 1 (MO) with the sequence TTG CAG GTC CTA CCT GGA GTC TGA G, the mismatched control morpholino (mmMO) has the sequence TTc CAG cTC CTA gCT GGA cTC TcA G (mismatched nucleotides in lower case). We injected 0.5 pmol of either matched or mismatched morpholino per embryo. RT-PCR to verify knockdown efficiency was carried out using primers CTG GGT ACC GCT TCT AGC A and CGG TCG CCT TTT TCC TTT T.

For gene overexpression experiments, we cloned full-length zebrafish *sfrp5* into pCS2+. We deleted the *dishevelled*, *egl-10*, and *pleckstrin* (DEP) domain of *dvl2* [42], [43] by combining PCR products of the *dvl2* N-terminus (aas 1–425) and C-terminus (aas 495–747) using overlapping PCR (for primer sequences and ZFIN and GenBank accession numbers, see Supporting Table S1). The zebrafish *chd-6xMyc* and *Xenopus laevis wnt11b* constructs were kind gifts from Drs. Fisher and Davidson [44]–[46]. Capped and polyadenylated mRNA was transcribed using mMessage Machine (Life Technologies) and injected into 1- to 2-cell embryos. Based on the experimental endpoint, we optimized the amount of *sfrp5* mRNA that we injected.

### *In situ* Hybridization and Immunohistochemistry

Whole-mount *in situ* hybridization was carried out as previously described [39]. For gene and primer information, including accession numbers, refer to Supporting Table S2. For confocal microscopy, outcrossed *Tg*(*Xla.Eef1a1:GFP*)*^s854^* embryos [hereafter referred to as gutGFP] were injected as above and processed as previously published [47]. Images were acquired on a Zeiss LSM700 confocal microscope and analyzed with ImageJ (US National Institutes of Health). Cell size was calculated by dividing the organ size by the number of GFP^+^ cells. Probabilities were calculated using Student’s *t*-test and boxplots generated using R (http://www.r-project.org/).

### *Tg(hs:mCherry,wnt2bb)* Transgenic Fish Line and Heat Shock Conditions

To generate the *Tg*(*hsp70l:mCherry-T2A-wnt2bb,cryaa:ECFP*)*^pt603^* line [hereafter referred to as *Tg*(*hs:mCherry,wnt2bb*)], an injection construct was created using multisite Gateway technology (Life Technologies) with the Tol2 destination vector *pDestTol2pA2AC* containing the *cryaa:eCFP* construct [48], [49]. For primer and gene information, please see Supporting Table S3. The construct was microinjected together with *tol2* mRNA into wild type 1-cell embryos as previously described [48]. Multiple transgenic lines were established, and the best representative transgenic line was used for all experiments.

Heterozygous transgenic fish were outcrossed to AB* wild type fish, injected with either 100 pg *eGFP* or 100 pg *sfrp5* mRNA at the 1- to 2-cell stage, heat-shocked at the 18 somite-stage for 40 min at 38.5°C, and sorted into *wnt2bb* overexpressing (mCherry^+^) or control embryos (mCherry^–^). Embryos were analyzed by *in situ* hybridization at 48 hpf as described in the text.

### Chd Stability Assay

N-terminal, epitope-tagged forms of *tll1*, *sfrp2*, and *sfrp5* were made by combinatorial use of overlapping PCR. *eGFP* was amplified from plasmid pEGFP1 (Clontech). The 3x FLAG tag (Sigma-Aldrich) was amplified by PCR. For efficient secretion of zebrafish proteins in 293T cells, we used the signal peptide from human insulin (OpenBiosystems). Zebrafish cDNAs encoding *tll1*, *sfrp2*, and *sfrp5*, each lacking the predicted signal sequences, were amplified by PCR (see Supporting Table S3 for accession numbers, primer sequences, and regions amplified). Fragments were then combined by overlapping PCR to create the following constructs: insulin signal peptide followed by 3x FLAG and *tll1*; and insulin signal peptide followed by *GFP* and either *sfrp2* or *sfrp5*. Complete coding sequences were subcloned into pGEM-T Easy (Promega), sequence verified, and moved into pCS2+.

293T cells were transfected with a single plasmid encoding a single tagged gene and pCS2+ mCherry as a transfection control using FuGene HD (Roche) per manufacturer’s instructions at a ratio of 2.5 µg DNA to 5 µl FuGene reagent per each well of a 6-well plate. Transfected cells were grown in serum free media consisting of a 1∶1:1 mix of DMEM, IMEM, and F12 (Hyclone). After 72 hours, conditioned media containing secreted proteins were collected and frozen at −80°C. Protease digests were carried out by combining conditioned media and incubating at 37°C for 5 hrs. The volume of conditioned media from *chd-6xMyc* or *3xFLAG-tll1* transfected cells was kept constant. Conditioned media from *eGFP-sfrp2* or *eGFP-sfrp5* transfected cells was doubled or tripled to increase the concentration of Sfrp2 or Sfrp5. Digests were run on Tris-Tricine gels (Bio-Rad) and transferred to PVDF membrane. Membranes were probed with a monoclonal antibody against C-MYC (Roche, Cat. Nr. 11667149001), monoclonal antibody M2 against the FLAG-tag (Sigma-Aldrich, Cat. Nr. F3165) or with a polyclonal antibody against the N-terminus of eGFP (Sigma-Aldrich, Cat. Nr. G1544). All primary antibodies were used at a dilution of 1∶500. Secondary antibodies were AP-conjugated anti-mouse antibodies (Southern Biotechnology, Cat. Nr. 1031-04, Dilution 1∶5,000) and HRP-conjugated anti-rabbit antibody (eBiosciences TrueBlot Ultra, Cat. Nr. 18-8817-30, Dilution 1∶20,000). AP-conjugated secondary antibodies were detected by CDP* (Perkin-Elmer), and HRP-conjugated secondary antibodies were detected using SuperSignal West Pico (Thermo Scientific).

## Results

### *sfrp5* Expression Profile in Developing Zebrafish Embryos

**Figure 1:**
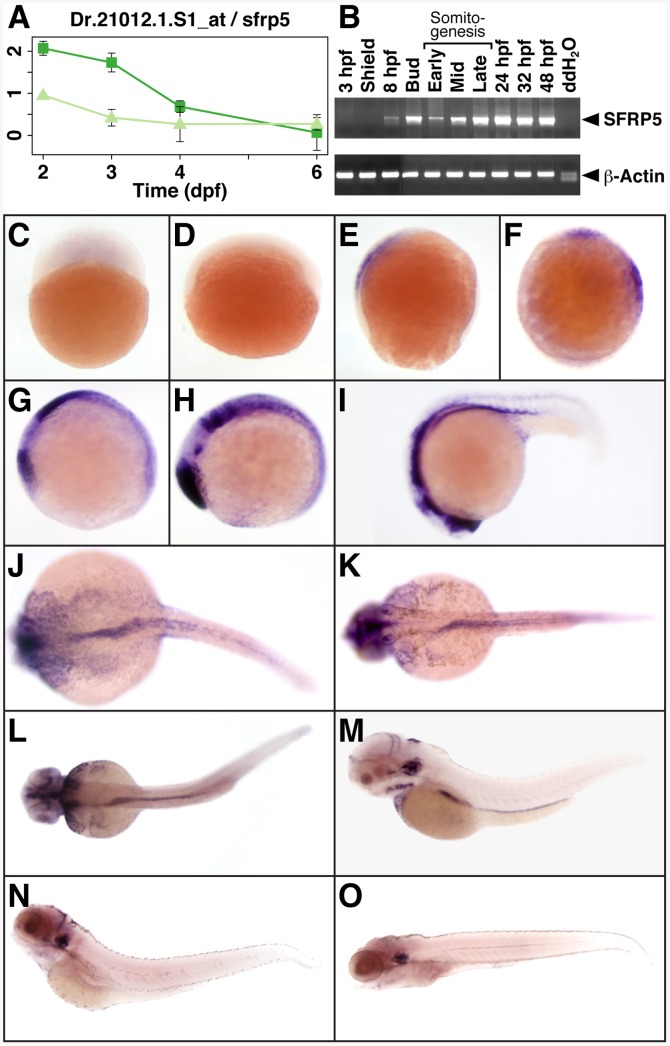
Expression profile of *sfrp5*. **A)** Expression level of *sfrp5* as measured by probeset Dr.21012.1.S1 in GI tissue (dark green squares) and non-GI tissue (light green triangles) from 2 through 6 dpf (for details, see [39]). **B)** Expression of *sfrp5* and β-actin by RT-PCR of total RNA isolated at indicated time points. **C–O)** Whole-mount *in situ* hybridization showing *sfrp5* expression in zebrafish embryos at 3 hpf (**C**), shield stage (**D**), 8 hpf (**E**), bud stage (**F**), early (**G**), mid (**H**), and late somitogenesis (**I**), 24 hpf (**J**), 32 hpf (**K**), 2 dpf (**L**), 3 dpf (**M**), 4 dpf (**N**), and 6 dpf (**O**). Lateral views with animal pole to the top (**C**–**F**) or with anterior to the left (**G**–**I**, **M**–**O**). Dorsal view with anterior to the left (**J**–**L**).

While analyzing the transcriptome of the developing GI tract in zebrafish, we identified *sfrp5* as a gene with an expression profile that suggested an important role in GI organogenesis [39]. We especially noted high levels of transcript during early stages of organogenesis, decreasing to background levels once the expression of genes associated with organ function increases (Fig. 1A). We confirmed the expression profile by RT-PCR on RNA from whole embryos (Fig. 1B) and *in situ* hybridization (Fig. 1C–O). *sfrp5* is not deposited maternally or expressed very early in development (Fig. 1B–D). Its expression is first detected at 8 hours post fertilization (hpf) by both RT-PCR and *in situ* hybridization, localizing to the anterior neural plate (Fig. 1B, E–L). Expression remains detectable until 6 days post fertilization (dpf), the last time point analyzed. Expression in the endodermal rod is apparent from 24 hpf through 3 dpf (Fig. 1J–M). A few embryos still express *sfrp5* in the intestine at 4 dpf (data not shown), but most do not (Fig. 1N). Expression of *sfrp5* remains strong in otoliths even at 4 and 6 dpf, while its expression is largely diminished in the rest of the larval tissues at this stage (Fig. 1N, O). Our data are in good agreement with the expression profile previously published from early somitogenesis up until 48 hpf (Fig. 1F–L) [25].

### Early Developmental Phenotype

We injected 0.5 pmol of a morpholino targeted to the first splice site of *sfrp5* or 140 pg of *sfrp5* mRNA into 1- to 2-cell stage embryos to determine the role of *sfrp5* in early development and organogenesis. We noted that overexpression of *sfrp5*, but not Sfrp5 knockdown, caused early developmental defects.

**Figure 2:**
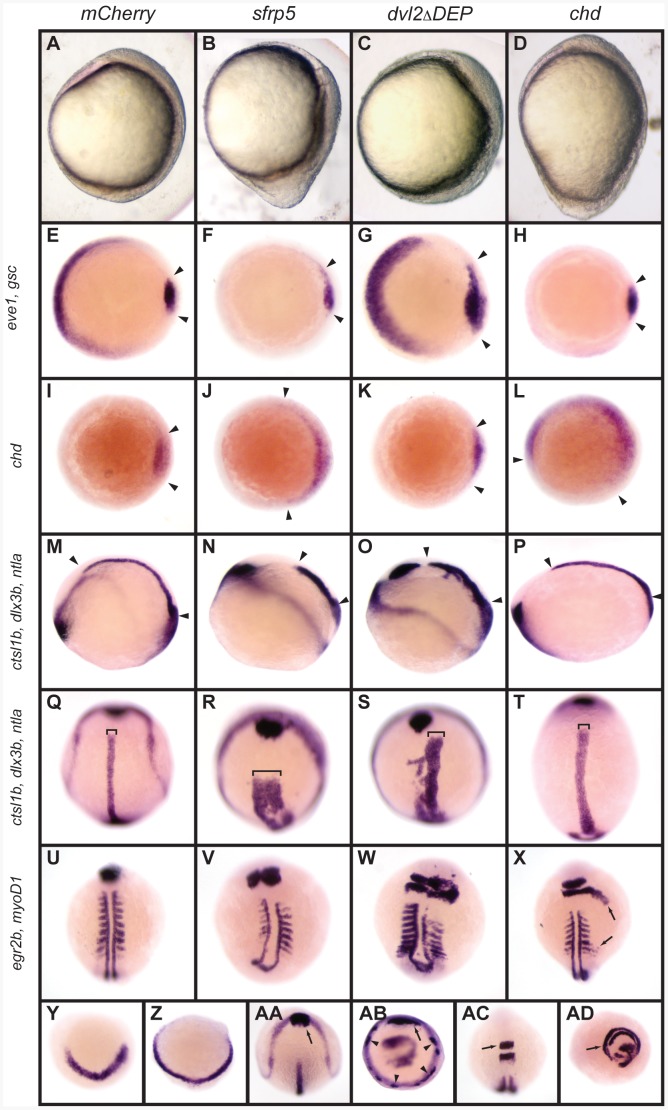
Overexpression of *sfrp5* disrupts gastrulation. Embryos were injected with 100 pg *mCherry* as control (A, E, I, M, Q, U, Y, AA, AC), 140 pg *sfrp5* (B, F, J, N, R, V, Z, AB, AD), 150 pg *dvl2*Δ*DEP* (C, G, K, O, S, W), or 50 pg *chd* (D, H, L, P, T, X). **A**–**D)** Morphology of injected embryos injected at early somitogenesis, lateral view with dorsal side to right. **E**–**AD)** Whole-mount *in situ* hybridization of injected embryos. **E**–**H)** Animal pole view with dorsal side to the right of embryos stained with *eve1* and *gsc* probes at shield stage. Arrowheads demarcate *gsc* staining. **I**–**L**) Animal pole view with dorsal side to the right of embryos stained with *chd*, demarcated by arrowheads, at shield stage. **M**–**T**) Early somitogenesis embryos stained with probes against *ctsl1b*, *dlx3b*, and *ntla.* M–P: Lateral view with dorsal to top. Arrowheads mark length of notochord. Q–T: dorsal view with anterior to top. Brackets show notochord width. **U**–**X**) Mid-somitogenesis embryos stained with *egr2b* and *myoD1*. Dorsal view with anterior to top. In X, arrows mark radialization of *egfr2b* and *myoD1* staining. **Y**, **Z**) Bud-stage embryos hybridized with probe against *her5*; anterior view, dorsal to bottom. **AA–AB**) Early somitogenesis embryos hybridized with probes against *ctsl1b*, *dlx3b*, and *ntla*. Arrow: normal *ctsl1b* staining. Arrowhead: ectopic *ctsl1b* staining. AA: Dorsal view, anterior to top. AB: Ventral view, dorsal to top. **AC**–**AD**) Mid-somitogenesis embryos hybridized with probes against *egr2b* and *myoD1*. AC: Dorsal view, anterior to top. AD: ventral view, dorsal to top. Arrows point to rhombomere 3.

The early developmental defects observed in embryos overexpressing *sfrp5* appear to combine features of both dorsalization and convergence and extension (CE) defects (Fig. 2A–D). At bud stage, *sfrp5* injected embryos are shortened along the antero-posterior axis and the bud extends caudally off the yolk (Fig. 2B). To better categorize the defects in *sfrp5* overexpressing embryos, we compared them to embryos dorsalized by overexpression of *chordin* (*chd*) [50], [51] and to embryos with defective CE by overexpression of a mutant form of *dishevelled*, which lacks the DEP domain (*dvl2*Δ*DEP*) and acts as a dominant-negative inhibitor of non-canonical Wnt signaling [42], [43]. The axial shortening of *sfrp5* overexpressing embryos is comparable to the axial shortening in embryos with CE defects (Fig. 2C), while the thickening of the bud is seen in embryos dorsalized by overexpression of *chd* (Fig. 2D). We analyzed the apparent dorsalization phenotype of *sfrp5* overexpression by *in situ* hybridization against *even-skipped1* (*eve1*; Fig. 2E–H), *goosecoid* (*gsc*, Fig. 2E–H), and *chordin* (*chd,* Fig. 2I–L); molecular markers for ventral (*eve1*) and dorsal embryonic domains (*gsc*, *chd*) [52]–[54]. Overexpression of *sfrp5* resulted in a marked decrease in the ventral marker *eve1* (Fig. 2F), which is also reduced in dorsalized embryos (Fig. 2H). Expansion of *gsc* staining was only seen in embryos with CE defects (Fig. 2F, G), but not in embryos dorsalized by overexpression of *chd* (Fig. 2H), consistent with existing data showing that *gsc* expression is unaffected by increased Chd levels [50]. Unlike *gsc*, the *chd* expression domain increased in embryos overexpressing either *sfrp5* or *chd* (Fig. 2J, L), but was unaffected in control embryos and those with CE defects (Fig. 2I, K). Taken together, these data show that as early as shield stage, dorsoventral patterning is disrupted in embryos overexpressing *sfrp5*.

We examined embryonic patterning in early somitogenesis by *in situ* hybridization using a mix of three probes. One of the probes was *ctsl1b*, formerly *hgg1*, a marker of the prechordal plate. Additionally, the mix contained *dlx3b*, a marker of the neural border, and *ntla*, a marker of the notochord and tail bud [55]. Shortening of the notochord was revealed in *sfrp5* overexpressing embryos by *ntla* expression (Fig. 2N). This defect was also seen in embryos injected with *dvl2*Δ*DEP* and had CE defects, but not in embryos dorsalized by *chd* overexpression (Fig. 2M–P). Additionally, we observed a thickening of the notochord in *sfrp5* overexpressing embryos (Fig. 2Q–T, compare bracket). While also present to some extent in dorsalized embryos (Fig. 2T), the effect is most pronounced in embryos overexpressing *dvl2*Δ*DEP* (Fig. 2S) and embryos overexpressing *sfrp5* (Fig. 2R). In embryos overexpressing *sfrp5* or *dvl2*Δ*DEP*, it also appeared that axial mesodermal cells expressing *ntla* did not coalesce at the midline, as illustrated by the multiple cells that failed to converge on the midline (Fig. 2S). In *sfrp5* overexpressing embryos, some cells at the center of the notochord did not stain positive for *ntla*. Such cells were not observed in uninjected or control embryos (Fig. 2Q, R). Additionally, in many embryos overexpressing either *sfrp5* or *dvl2*Δ*DEP* the notochord undulated or had pronounced kinks (Fig. S1).

Failure to migrate to the midline was seen in some embryos overexpressing either *sfrp5* or *dvl2*Δ*DEP* (Fig. 2V, W) by incomplete fusion of the four fields of *egr2b* staining (formerly *krox20*) [56] to two bands, one each at rhombomeres 3 and 5, and a wider space between the adaxial *myoD1* staining (Fig. 2V, W), when compared to *mCherry* or *chd* injected embryos (Fig. 2U, X). Additionally, the somites were often of variable length and misaligned along the anterioposterior axis, as shown by *myoD1* staining [57]. Overexpression of *chd* caused radialization of both *egr2b* and *myoD1* staining in a few embryos and pushed *egr2b* and *myoD1* positive cells posteriorly (Fig. 2X), as has also been observed in other dorsalized embryos [50], [58], [59]. *sfrp5* embryos displayed radialization as well: expression of *her5*, a marker of the midbrain primordium [60], was radialized in some embryos (Fig. 2Y, Z), *ctsl1b* was sometimes seen staining the entire embryonic perimeter in non-contiguous patches (Fig. 2AA, AB), and cells expressing *egr2b* were also seen encircling the entire embryo in some severely dorsalized animals after overexpression of *sfrp5* (Fig. 2AC, AD). We conclude that embryos overexpressing *sfrp5* show defects in both CE and dorsoventral patterning.

### Sfrp5 Inhibits Non-canonical Wnt Signaling and the Protease Tolloid

**Figure 3:**
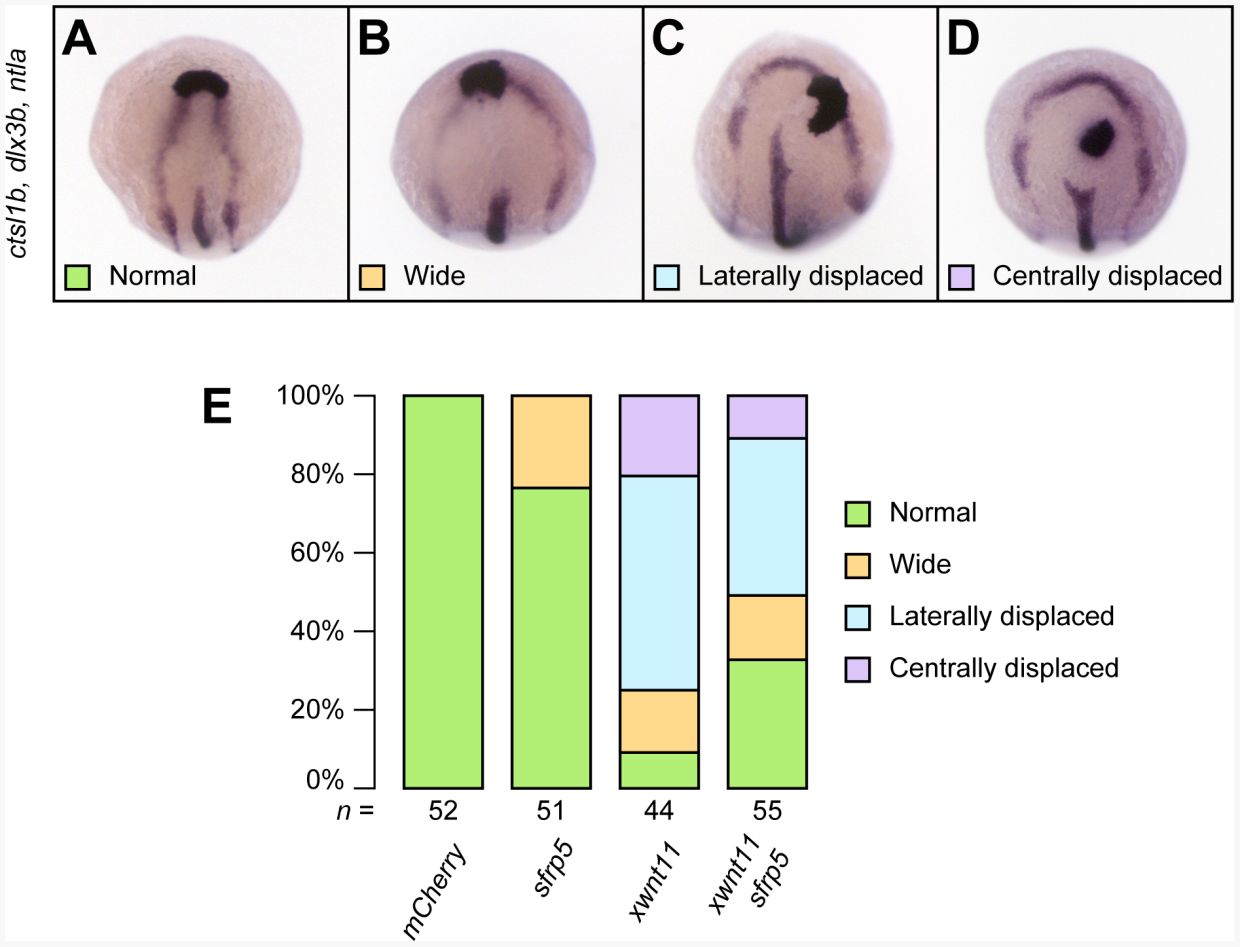
*sfrp5* overexpression inhibits non-canonical Wnt signaling. Embryos injected with 200 pg *mCherry*, 140 pg *sfrp5*, and 100 pg *wnt11b* from *Xenopus laevis* alone and in combination as indicated. **A**–**D**) 4-somite embryos were processed by *in situ* hybridization with probes against *ctsl1b*, *dlx3b*, and *ntla*. Dorsal view with anterior towards the top. The neural plate was scored as normal (**A**), widened (**B**), or widened with a laterally (**C**) or centrally (**D**) displaced prechordal plate. **E**) Bar chart showing percentage of different phenotypic classes for each treatment. The total number of embryos analyzed per treatment is shown under each column.

The observed CE defects during gastrulation in *sfrp5* overexpressing embryos were similar to those seen in embryos overexpressing *dvl2*Δ*DEP* and are consistent with inhibition of non-canonical Wnt signaling, but Sfrp5-mediated inhibition of non-canonical Wnt signaling had not been demonstrated in zebrafish before. To show that zebrafish Sfrp5 can inhibit non-canonical Wnt signaling, we overexpressed *Xenopus wnt11b*, an ortholog of zebrafish *wnt11*, in zebrafish embryos and observed defects similar to those reported with overexpression of zebrafish *wnt11* [61], specifically the widened neural plate and notochord as well as a prechordal plate that was shifted laterally or posteriorly, away from the edge of the neural plate (Fig. 3, A–D). Co-expression of zebrafish *sfrp5* ameliorated the phenotype of *wnt11b* overexpression and resulted in more normal embryos (Fig. 3E), indicating that Sfrp5 inhibited Wnt11b-mediated non-canonical Wnt signaling.

Our finding that Sfrp5 can modulate dorsoventral patterning in addition to inhibition of non-canonical Wnt signaling suggests that Sfrp5 may regulate other signaling pathways as well. One candidate pathway is the BMP signal transduction pathway, as the Sfrp family members Sfrp2, Sizzled, and Crescent have been shown to inhibit Tolloid (Tll1), a protease inactivating Chordin (Chd), an inhibitor of BMP signaling. BMP signaling plays crucial roles in dorsoventral patterning and is therefore an attractive candidate signaling pathway to explain defects in dorsoventral patterning in embryos overexpressing *sfrp5* [9], [11], [12]. *tll1* inactivation by mutation or knockdown dorsalizes zebrafish embryos [58], [59], [62]. We reasoned that since *sfrp5* is closely related to s*frp2* [25], excess Sfrp5 might inhibit Tll1 and stabilize Chd, hence causing dorsalization, analogous to what is observed for Sfrp2 in *Xenopus* and for Sizzled in *Xenopus* and zebrafish [9], [11]. We tested this hypothesis in two complementary ways. First, we co-injected combinations of *chd*, *tll1* and *sfrp2* or *sfrp5* and analyzed dorsoventral patterning at the 4 somite stage. Secondly, we biochemically tested Sfrp5 for its ability to inhibit Tll1-mediated proteolysis of Chd *in vitro*.

**Figure 4:**
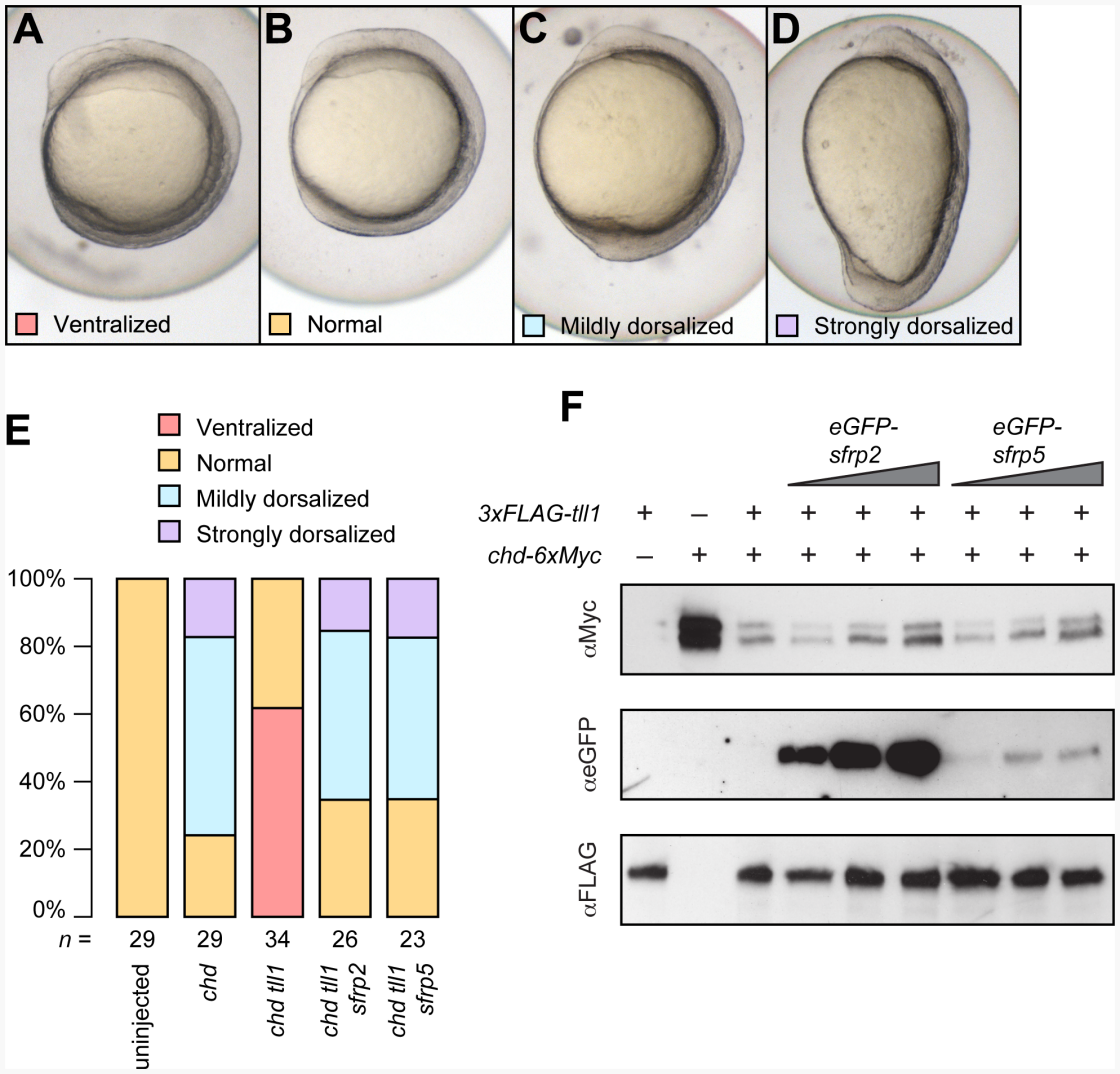
Sfrp5 inhibits the Tll1 protease. Embryos were injected with combinations of 50 pg *chd*, 200 pg *tll1*, 150 pg *sfrp2*, and 125 pg *sfrp5.* At the 4 somite stage, embryos were classified as ventralized (**A**), normal (**B**), mildly dorsalized (corresponding to C1– C3; **C**) or strongly dorsalized (C4– C5; **D**) [59] and the results plotted (**E**). The numbers under each bar represent the number of embryos analyzed per treatment. **F**) Conditioned media from singly transfected 293T cells were combined as indicated, incubated, and analyzed by Western blotting. Volume of conditioned media from *tll1* and *chd* transfected cells was kept constant when used, but volume of conditioned media from *sfrp2* or *sfrp5* transfected cells was doubled or tripled as indicated.

To test whether Sfrp5 can inhibit Tll1 *in vivo*, we first injected *chd-6xMyc* with and without *3xFLAG-tll1* into 1- to 2- cell stage embryos. We categorized embryos into severely dorsalized (classes C4 and C5), mildly dorsalized (classes C1– C3), normal, and ventralized embryos (Fig. 4A–D) [59], [63]. As expected, overexpression of *chd* dorsalized most embryos, but coinjection of *tll1* countered the dorsalization activity of *chd* and resulted in a high percentage of ventralized embryos (Fig. 4E). Additional overexpression of either *sfrp2* or *sfrp5* offset the activity of *tll1* and resulted in similar levels of dorsalization compared with injection of *chd* alone (Fig. 4E). Therefore, in live embryos, *sfrp2* and *sfrp5* could inhibit the ventralization brought about by Tll1.

To show that Sfrp5 could inhibit Tll1 *in vitro*, we singly transfected 293T cells with epitope-tagged forms of *chd*, *tll1*, *sfrp5*, and *sfrp2* as a control, combined conditioned media, and assayed Tll1 inhibition by Chd stabilization (Fig. 4F). As had been reported previously, addition of zebrafish Tll1 cleaved zebrafish Chd [11], [64]. We show here that high levels of zebrafish Sfrp2, as expected, inhibit Tll1 function [9]. Sfrp5 was capable of inhibiting Tll1 as well, but appeared to be more potent, based on signal intensity of the eGFP tag (Fig. 4F). In addition, we note that low levels of either Sfrp2 or Sfrp5 appear to promote Tll1-mediated Chd proteolysis, suggesting that both zebrafish Sfrp2 and Sfrp5 function in a biphasic manner. While this was unexpected, another Sfrp protein, Sfrp1, sets a precedent for biphasic function in *Drosophila* tissue culture cells; low concentrations of Sfrp1 enhance Wingless signaling, but high concentrations inhibit it [27]. Our results showing that *sfrp5* overexpression can counter ventralization mediated by Tll1 in live embryos and that high concentrations of Sfrp5 can inhibit Tll1-mediated proteolysis of Chd provide an avenue to explain dorsalization of embryos overexpressing *sfrp5.*

### Overexpression and Knockdown of sfrp5 Affect Liver Formation

**Figure 5:**
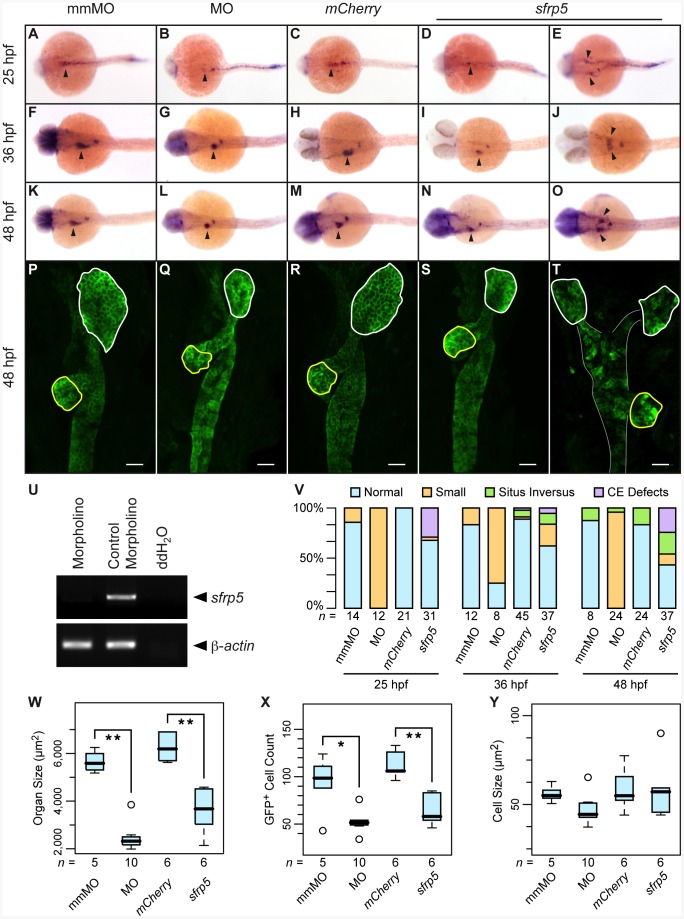
Sfrp5 regulates hepatoblast formation in zebrafish. Embryos were injected with 0.5 pmol morpholino against *sfrp5* (MO; B, G, L, Q), 0.5 pmol of the control morpholino (mmMO; A, F, K, P), 100 pg *mCherry* mRNA (C, H, M, R), or 100 pg *sfrp5* mRNA (D, E, I, J, N, O, S, T). **A**–**O**) Whole-mount *in situ* hybridization with a probe against *hhex* staining embryos at 25 hpf (A–E), 36 hpf (F–J), and 48 hpf (K–O). Dorsal view, anterior to the left. Arrowheads point to the hepatoblast. **P**–**T**) Confocal microscopy of injected gutGFP embryos. Ventral view with anterior to top. Liver is outlined in white, pancreas in yellow. The scale bar is equal to 25 µm. **U**) RT-PCR of morpholino and control injected embryos with primer pairs detecting *sfrp5* or β-actin. **V**) Bar chart representing distribution of normal and abnormal embryos processed by *in situ* hybridization, with representative samples shown in A–O. **W**) Boxplot showing liver size distribution in injected embryos. **: *p*<0.001. **X**) Boxplot showing distribution of the number of GFP^+^ liver cells. *: *p*<0.05, **: *p*<0.01. **Y**) Boxplot showing liver cell size distribution. Numbers below each column or boxplot show how many embryos were analyzed.

Because of the strong expression of *sfrp5* in the endoderm (Fig. 1), we wanted to assay the effect of modulating Sfrp5 levels on the overall development of the GI tract. We injected embryos with a morpholino against the exon 1– intron 1 boundary of the *sfrp5* gene (MO) or a mismatched morpholino (mmMO) as control. We showed by RT-PCR that *sfrp5* mRNA levels were still absent 30 hpf after injection of 0.5 pmol of morpholino (Fig. 5U). To examine the effects of *sfrp5* overexpression, we injected 100 pg of either *mCherry* or *sfrp5* mRNA. Because of the importance of Wnt signaling in liver development in zebrafish [41], [65], [66], we focused on the impact of modulating Sfrp5 levels on the development of the hepatoblast, as shown by staining with *hhex* [67], [68]. We found that already at 25 hpf, both morpholino and *sfrp5* injected embryos showed abnormalities (Fig. 5A–E, V). Injection of the *sfrp5* morpholino reduced the size of the hepatoblast compared to mismatch controls (Fig. 5A, B, V). A smaller number of embryos overexpressing *sfrp5* also showed abnormalities, such as a smaller hepatoblast and endoderm that failed to coalesce into a single rod (Fig. 5D, E). We observed similar results at both 36 and 48 hpf. Almost all morpholino-injected embryos displayed a reduced hepatoblast and also a smaller dorsal pancreatic bud (Fig. 5F, G, K, L). Embryos overexpressing *sfrp5* also showed a smaller hepatoblast and a smaller pancreas compared to control-injected embryos (Fig. 5H, I, M, N), but fewer embryos were affected compared to morpholino-injected embryos (Fig. 5V) and the effect was generally less severe (Fig. 5W). In embryos overexpressing *sfrp5*, we also observed a number of embryos in which endodermal cells failed to coalesce into a single endoderm (Fig. 5E, J, O, T) and embryos with *situs inversus*, in which the organs form on the wrong side of the embryo (data not shown).

For a more detailed assessment of changes in organ morphology at 48 hpf, we analyzed gutGFP embryos injected with morpholino or mRNA. gutGFP transgenic embryos express GFP in the developing digestive system. Liver, pancreas, and intestine are clearly visible by confocal microscopy at 48 hpf [69]. At that time, embryos injected with morpholino, but not mismatch morpholino, showed a less organized and much smaller liver and smaller pancreas (white and yellow outlines; Fig. 5P, Q, W). As we observed in the embryos stained for *hhex*, injection of *sfrp5* mRNA caused a decrease in both pancreas and liver size that was smaller than the decrease seen in morpholino-injected embryos (Fig. 5R, S, W and Fig. S2). While the decrease in liver size in both knockdown and overexpression embryos was statistically significant, the decrease in pancreas size was not (Fig. 5W, Fig. S2). The decrease in organ size was mainly due to a statistically significant reduction in GFP^+^ cell number (Fig. 5X), the average size of a liver cell did not change significantly (Fig. 5Y). We observed few embryos with disordered endoderm and an apparent duplication of liver structures and a pancreas that is on the left side of the embryo, rather than the usual right side (ventral view; Fig. 5T), similar to embryos in which endodermal cells failed to coalesce at the midline as shown by *hhex* staining (Fig. 5E, J, O).

**Figure 6:**
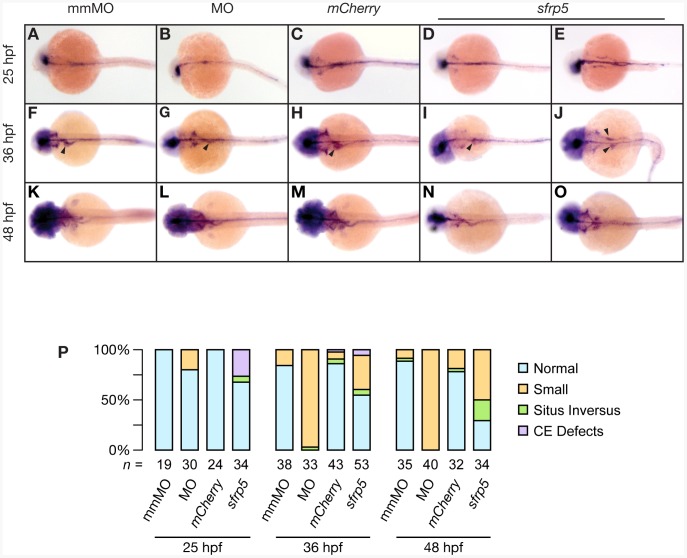
Endodermal defects in embryos with altered levels of Sfrp5. Embryos were injected with 0.5 pmol morpholino against *sfrp5* (MO; panels B, G, L), 0.5 pmol of the control morpholino (mmMO; panels A, F, K), 100 pg *mCherry* mRNA (C, H, M), or 100 pg *sfrp5* mRNA (D, E, I, J, N, O). **A**–**O**) Whole-mount *in situ* hybridization with a probe against *foxa1* staining embryos at 25 hpf (A–E), 36 hpf (F–J), and 48 hpf (K–O). Dorsal view, anterior to the left. Arrowheads point to the hepatoblast (F–J). **P**) Bar chart representing distribution of normal and abnormal embryos processed by *in situ* hybridization, with representative samples shown in A–O. Below each column, numbers of embryos analyzed.

We utilized the pan-endodermal marker *foxa1* [70] to determine any endodermal abnormalities in addition to the defects seen in the hepatoblast, as shown by *hhex* staining (Fig. 4). We found that our results with *foxa1* were consistent with the results using *hhex.* At 25, 36, and 48 hpf, embryos injected with the morpholino or *sfrp5* mRNA displayed a smaller endoderm compared with controls and morpholino injected embryos were more severely affected than embryos overexpressing *sfrp5* mRNA (Fig. 6). In addition to hepatic defects, the pancreatic buds and the thickness of the intestine appeared to be smaller as well. However, unlike liver size, differences in pancreas size were not statistically significant, though they too suggested a predominant effect on cell number, rather than cell size (Fig. S2). Some embryos overexpressing *sfrp5* showed two hepatic buds and possibly two intestinal rods, suggesting that endodermal precursor cells had failed to coalesce at the midline during gastrulation (Fig. 6E, J, O), consistent with the CE defects observed during early development (for example, Fig. 2R). Overall, we found that modulation of Sfrp5 protein levels resulted in changes throughout the developing endoderm, with the liver being most severely affected.

### Alteration of Sfrp5 Levels Affect Endodermal Organs up to 3 dpf

**Figure 7:**
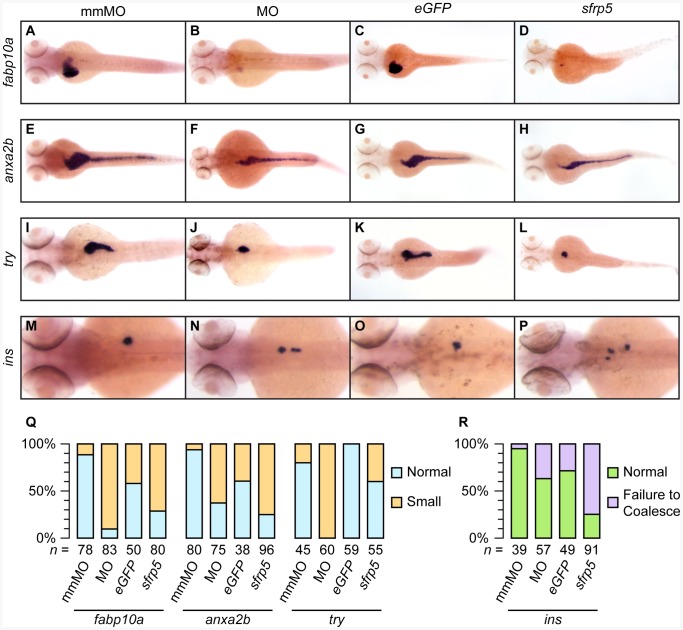
Modulation of *sfrp5* expression causes defects in gastrointestinal development. **A** ****–**P)** Dorsal views of 3 dpf old embryos stained with probes against *fabp10a* (**A**–**D**), *anxa2b* (**E**–**H**), *try* (**I**–**L**), and *ins* (**M**–**O**). Embryos were injected with 0.5 pmol mismatch morpholino (A, E, I, M), 0.5 pmol morpholino against *sfrp5* (B, F, J, N), 100 pg *eGFP* mRNA (C, G, K, O) or 50 pg *sfrp5* mRNA (D, H, L, P). **Q**) Chart summarizing *in situ* results (A–L), showing percentages of normal or small GI organs. **R**) Chart summarizing *in situ* results (M–P), showing the percentage of larvae in which *ins* ^+^ cells failed to coalesce. The total number of analyzed embryos per treatment is shown below each column.

To determine if defects in endodermal organ specification might also result in defects in organ specification, we chose four markers of mature organ function: *fatty acid binding protein 10a* (*fabp10a*) for liver, *annexin A2b* (*anxa2b*) for intestine, *trypsin* (*try*) for exocrine pancreas, and *preproinsulin* (*ins*) for endocrine pancreas. We injected 1–2 cell embryos with either a morpholino against *sfrp5* or 50 pg of *sfrp5* mRNA. Analysis of morpholino injected embryos at 3 dpf by whole mount *in situ* hybridization showed that the size of liver, pancreas, and intestine was markedly decreased (Fig. 7). Similar to the earlier time points of 25 and 36 hpf, the mRNA injected embryos showed a reduction in organ size, but the reduction was smaller and, with the exception of *anxa2b*, occurred less frequently (Fig. 7Q). We found evidence for smaller liver (Fig. 7A–D, Q), intestine (Fig. 7E–H, Q), and exocrine pancreas (Fig. 7I–L, Q) in both knockdown and overexpression embryos.

We noticed, however, that in the case of the endocrine pancreas, as shown by *ins* staining, many embryos overexpressing *sfrp5* had multiple, distinct groups of *ins*-positive cells scattered across the trunk of the animal, from left to right side, approximately at the level of the anterior endoderm (Fig. 7O, P). These groups of cells appeared to have failed to coalesce into a single endocrine pancreas, a phenotype that is also observed in animals in which Wnt5 had been removed by morpholino knockdown, thus interfering with non-canonical Wnt signaling [71]. While this phenotype was also present in morpholino-injected embryos to some extent, it was much less pronounced and less frequent (Fig. 7M, N, and R).

### Sfrp5-Mediated Inhibition of Canonical Wnt Signaling

**Figure 8:**
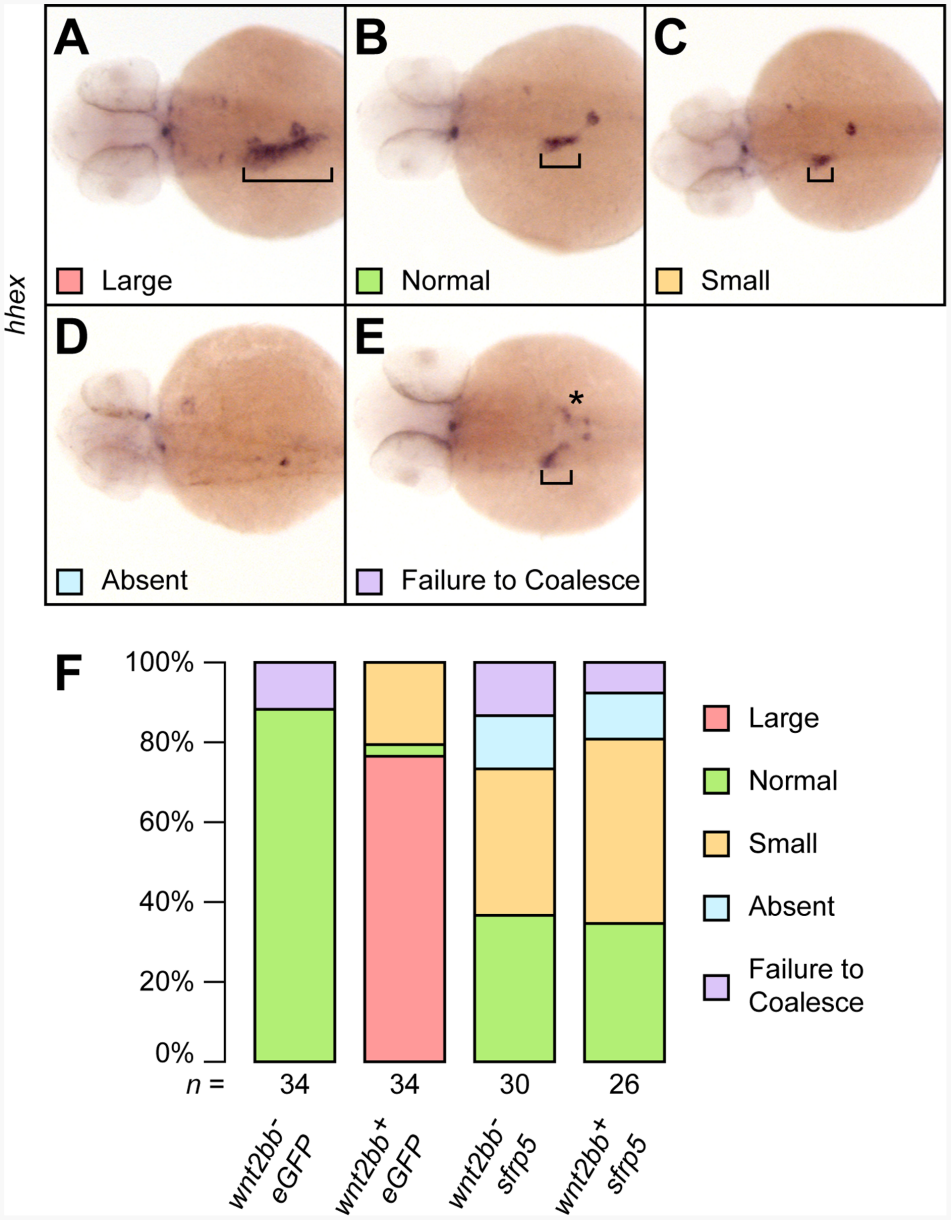
Overexpression of *sfrp5* inhibits canonical Wnt signaling. Clutches of 1- to 2-cell stage embryos obtained from an outcross of *Tg*(*hs:mCherry,wnt2bb*) heterozygotes with wild type fish were injected with either 100 pg *eGFP* or 100 pg *sfrp5* and sorted based on expression of *mCherry* after heat shock. **A**–**D**) 48 hpf embryos were analyzed for liver formation by *in situ* hybridization with *hhex* and categorized as having an enlarged (**A**), normal (**B**), or small liver (**C**). We also observed some embryos without apparent hepatoblast (**D**) or in which the hepatoblast failed to coalesce into a single field (**E**). Square brackets indicate the size of the hepatoblast, the asterisk in (D) mislocalized *hhex* positive cells. **F**) Bar chart showing the percentages of each category per treatment. The total number of analyzed embryos per treatment is shown below each column.

Overexpression of *sfrp5* in zebrafish has been shown to inhibit canonical Wnt-signaling mediated by Wnt8a [37]. Since canonical Wnt signaling, especially signaling downstream of Wnt2bb, is required for normal zebrafish liver development and proliferation [41], [65], [72], we wanted to test if *sfrp5* might be able to interfere with liver expansion mediated by overexpression of *wnt2bb*. We used a line of transgenic fish [*Tg*(*hs:mCherry,wnt2bb*)] expressing zebrafish *wnt2bb* under control of a heatshock promoter and crossed heterozygotes to wild type fish. Half of the resulting clutch was positive for the transgene and overexpressed *wnt2bb* after heatshock, the other half did not carry the transgene and served as negative control. We compared liver size at 48 hpf by *in situ* hybridization using the marker *hhex*. Embryos injected with *eGFP* and also overexpressing *wnt2bb* showed a marked expansion of liver size in almost all embryos, though we observed a small number of embryos with poorly defined, smaller livers (Fig. 8). Injection of *eGFP* in the absence of *wnt2bb* overexpression did not affect liver development. However, injection of *sfrp5* negatively affected liver development irrespective of overexpression of *wnt2bb*, significantly counteracting the liver expansion mediated by *wnt2bb* (Fig. 8E). Inhibition of *wnt2bb* is therefore a possible mechanism by which *sfrp5* reduces the size of the developing liver.

## Discussion

Wnt signaling plays a critical role in organismal development, organogenesis, and disease. In this work, we analyze the role of the secreted Wnt modulator Sfrp5 in development and organogenesis. It has been shown in *Xenopus* and mouse that Sfrp5 modulates both canonical and non-canonical Wnt signaling [16], [30] and we provide evidence that in zebrafish, Sfrp5 can also inhibit both pathways.

Inhibition of non-canonical Wnt signaling is likely to result in the observed CE defects in early development. Non-canonical Wnt signaling mediated by *wnt5* and *wnt11* plays an important role in CE movements, as zebrafish mutants for these genes have defects in CE [73]–[75]. Additionally, overexpression of *dvl2*Δ*DEP*, which specifically inhibits non-canonical Wnt signaling, results in CE defects that are similar to those observed by overexpression of *sfrp5* [42], [43]. In further support of this model, *Xenopus* Sfrp5 has been shown to bind both Wnt5b and Wnt11 *in vitro* [30].

Additionally, our results further support a model of Sfrp5 as inhibitor of both canonical and non-canonical Wnt signaling, as they add to previously published data showing that zebrafish Sfrp5 inhibits canonical Wnt signaling mediated by Wnt8a [37]. Additionally, in human, mouse, and *Xenopus*, Sfrp5 has been shown to inhibit canonical Wnt signaling [16], [30], [36], [38]. Our experiments showing inhibition of Wnt signaling mediated by *wnt2bb* are particularly instructive, as Wnt2bb is critical for normal liver expansion and zebrafish embryos mutant for *wnt2bb* have very small or absent livers [41], [65], [72].

**Figure 9:**
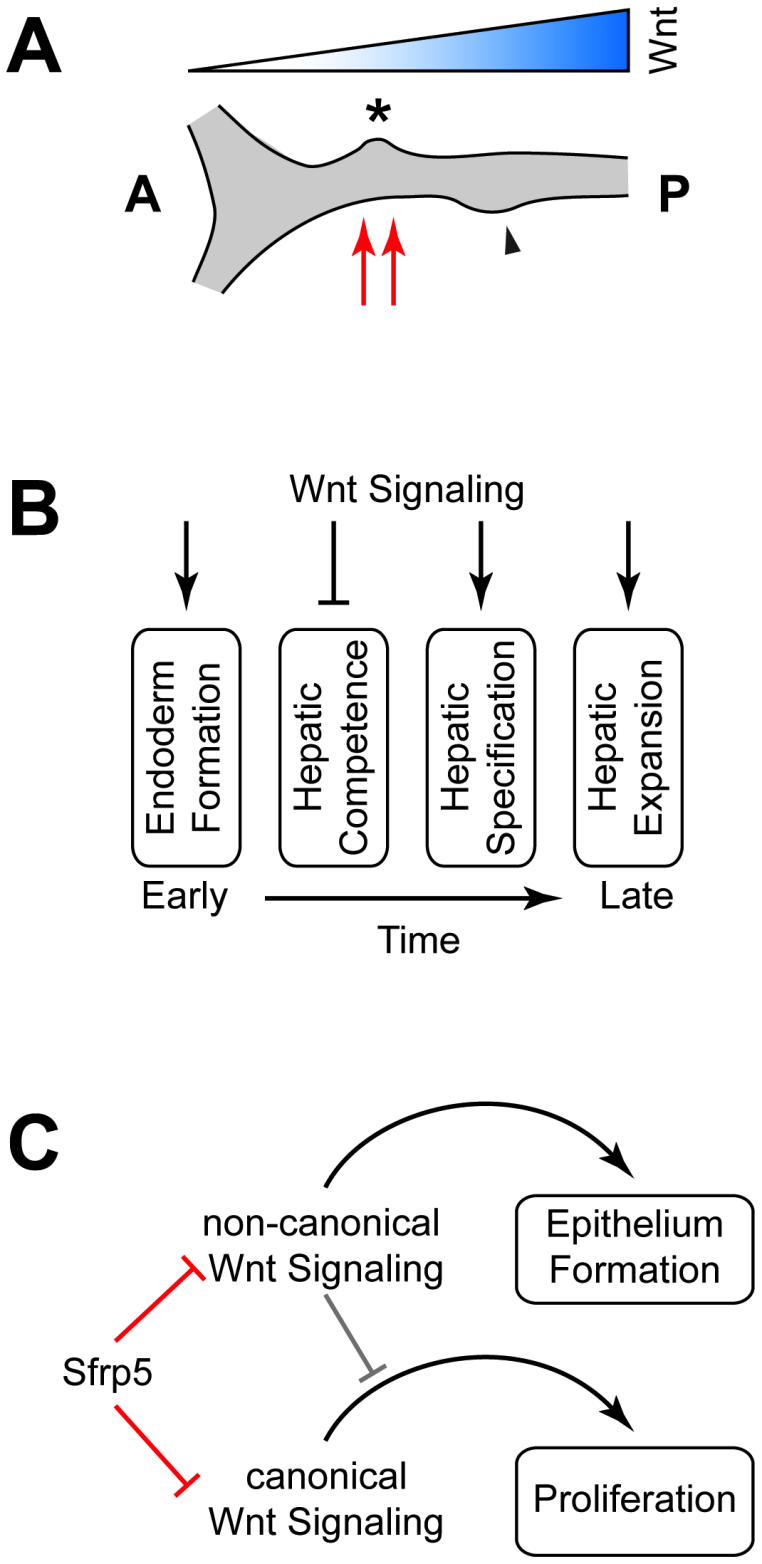
Three different models explaining the role of *sfrp5* in hepatic development. Three different models potentially explain the observed results. **A)** Wnt acts as a signaling gradient and any shift in the gradient by increasing or decreasing Sfrp5 levels misaligns the liver bud (asterisk) and pancreatic bud (black arrowhead) with mesodermal signals (red arrows). **B)** GI development requires both the presence of Wnt signaling and its absence at specific times during hepatic development (after [4]). **C)** Sfrp5 inhibits both canonical and non-canonical Wnt signaling and high levels of non-canonical Wnt signaling, possibly due to absence of Sfrp5, can inhibit canonical Wnt signaling.

How can both downregulation of Wnt signaling by *sfrp5* overexpression and upregulation of Wnt signaling by Sfrp5 knockdown result in similar endodermal defects, such as smaller GI organs? At least three mechanisms could explain our results, either alone or in combination (Fig. 9). First, different Wnt signaling thresholds may exist for specific biological responses [38], [65]. Research in multiple organisms shows that a gradient of Wnt signaling is important for endodermal patterning, with low levels of Wnt necessary anteriorly, and high levels of Wnt signaling required posteriorly [76]. If continued proliferation and expansion of the liver bud requires both the correct level of Wnt signaling and mesodermal signals [41], [65], [72], [77]–[79], both upregulation and downregulation of the Wnt gradient via overexpression or knockdown of Sfrp5 would misalign the mesodermal signal and the correct levels of Wnt signaling, reducing differentiation and/or proliferation and resulting in a smaller liver (Fig. 9A). Second, it is known that Wnt signaling plays multiple roles at different stages of hepatic development in many organisms, including mice, *Xenopus*, and zebrafish [4], [65], [80], [81]. Lack of Wnt signaling is required to establish hepatic competence, while presence of Wnt signaling is necessary for hepatoblast specification and expansion. Thus, both overexpression of *sfrp5* and Sfrp5 knockdown are expected to negatively affect liver development (Fig. 9B). The third possibility is that while overexpression of *sfrp5* can inhibit both canonical and non-canonical Wnt signaling, knockdown of Sfrp5 is expected to relieve inhibition of Wnt signaling – potentially leading to hepatic expansion. However, evidence shows that increased non-canonical Wnt signaling can inhibit canonical Wnt signaling [82], [83]. Therefore, both overexpression and knockdown of Sfrp5 could result in the same molecular defect, reduction in canonical Wnt signaling, either by directly preventing Wnt signals from interacting with their receptors in the extracellular space or by indirectly inhibiting canonical Wnt signaling (Fig. 9C).

Unlike overexpression of *sfrp5,* knockdown of Sfrp5 did not affect gastrulation, possibly due to the relatively late onset of *sfrp5* expression (Fig. 1), but also possibly due to redundancy between *sfrp5* and other earlier expressed *sfrps*, such as *sfrp1a* [25]. In this context, it is noteworthy that knockout of *Sfrp5* in mice had no observable defect on the expression profile of *Hhex*, on formation of the anterior visceral endoderm, or the axis [84]. However, triple-knockout mice lacking *Sfrp1*, *Sfrp2*, and *Sfrp5* were deficient in formation of the gut epithelium and displayed defects in CE movements that resulted in a shortened axis, a widened notochord and compressed, fused somites [16]–[18], indicating that these Sfrps have at least partially overlapping function in mice. Additionally, we note that the observed defects in the multiple knockout mice are similar to those we saw in embryos overexpressing *sfrp5*, just as we observed comparable results in *sfrp5* overexpression embryos and morphants on liver size. There are multiple examples in the literature showing that both a reduction and an increase in Wnt signaling, especially of non-canonical Wnt signaling, result in similar molecular and phenotypic defects. In chicks, both increasing and reducing non-canonical Wnt signaling affected gastrulation in similar ways [85] and in zebrafish, overexpression and reduction of w*nt11* result in similar gastrulation defects [61], [74].

In addition to convergent extension defects, embryos overexpressing Sfrp5 are dorsalized. Our results argue that Sfrp5 overexpression affects the stability of the BMP inhibitor Chordin by inhibiting Tolloid function, similar to the function of Sizzled, Sfrp2, and Crescent in zebrafish and *Xenopus* [9], [11], [12]. While overexpression of *sfrp5* could potentially dysregulate other early Wnt signaling events [86], our data show that Sfrp5 is capable of inhibiting Tll1 function both *in vivo* and *in vitro* and may decrease BMP signaling through stabilization of Chordin. These results support decreased BMP signaling as a possible explanation for the dorsalization phenotype in embryos overexpressing *sfrp5*.

Both NTR and CRD domains in SFRP proteins are rich in cysteines and form extensive disulfide bridges [26]. Recent findings have highlighted structural similarities between the disulfide bridge structure in the CRD of SFRPs and glypicans, such as Dally and Dally-like in *Drosophila* and the Glypicans GPC1 and GPC3 in *Homo sapiens*, suggesting that this particular arrangement of cysteines and their corresponding disulfide bridges is an evolutionarily conserved element that has been coopted by different proteins [87]. Glypicans are attached to the plasmamembrane and modulate many signaling pathways in the extracellular space, including Wnt, Hedgehog, TGF-β, and possibly FGF [88], [89]. Not surprisingly, they have been implicated in cancer and GPC3 is a candidate for targeted drug development against hepatocellular carcinomas [90]. Our results showing that Sfrp5 regulates the BMP signaling pathway in addition to Wnt signaling pathways further support a model where signal processing and cross-regulation of diverse pathways occurs in the extracellular matrix, emphasizing the importance of this space in development and disease.

## Supporting Information

Figure S1 **The notochord undulates and is kinked in** ***sfrp5*** **and** ***dvl2*** **Δ** ***DEP*** **injected embryos.** All embryos were processed by *in situ* hybridization using a cocktail of probes against *ctsl1b*, *dlx3b*, and *ntla* and are shown in dorsal view, anterior to top. **A)** Embryo injected with 200 pg of *mCherry* mRNA. **B)** Embryo injected with 50 pg *sfrp5* mRNA. **C)** Embryo injected with 140 pg *sfrp5* mRNA. **D)** Embryo injected with 150 pg *dvl2*Δ*DEP* mRNA. Arrows point to the notochord.
(TIF)

Figure S2 **Boxplots showing pancreas size distribution in embryos injected as in** **Figure 6** **.** **A**) Pancreas size in µm^2^. **B**) GFP^+^ cell number. **C**) Cell size in µm^2^. The total number of analyzed embryos per treatment is shown below each column.
(PDF)

Table S1 Primers used in cloning of injection vectors. The table shows the forward and reverse primers used in the cloning of the *sfrp5* and *dvl2*Δ*DEP* injection vectors along with the region amplified, the GenBank accession number, and ZFIN ID for the respective genes.
(PDF)

Table S2 Genes tested by *in situ* hybridization and primers used. This table shows the genes with their respective GenBank accession number and ZFIN ID that were used as probes for *in situ* hybridization. For probes that we generated for this manuscript, we also include the forward and reverse primers used.
(PDF)

Table S3 Primers used to create the transgenic injection construct and expression vectors for 293T transfection. This table shows the GenBank accession number and ZFIN ID number of genes used in the creation of the injection vector for the transgenic fish line *Tg*(*hs:mCherry,wnt2bb*) and for the vectors used in transfecting 293T cells. It also shows the forward and reverse primer used and the region amplified by the primers.
(PDF)
