# Correction: Sfrp5 Modulates Both Wnt and BMP Signaling and Regulates Gastrointestinal Organogensis in the Zebrafish, *Danio rerio*

**DOI:** 10.1371/annotation/024a206e-293f-4a95-a188-7838a2763266

**Published:** 2013-09-12

**Authors:** Carsten Stuckenholz, Lili Lu, Prakash C. Thakur, Tae-Young Choi, Donghun Shin, Nathan Bahary

The word "Organogenesis" was spelled incorrectly in the title. The correct title is: "Sfrp5 Modulates Both Wnt and BMP Signaling and Regulates Gastrointestinal Organogenesis in the Zebrafish, *Danio rerio*." 

The correct citation is: Stuckenholz C, Lu L, Thakur PC, Choi T-Y, Shin D, et al. (2013) Sfrp5 Modulates Both Wnt and BMP Signaling and Regulates Gastrointestinal Organogenesis in the Zebrafish, *Danio rerio*. PLoS ONE 8(4): e62470. doi:10.1371/journal.pone.0062470. 

