# Supplementary figures and images for: Sfrp5 Modulates Both Wnt and BMP Signaling and Regulates Gastrointestinal Organogensis in the Zebrafish, Danio rerio
Source: PLoS One. 2013 Apr 29;8(4):e62470. doi: 10.1371/journal.pone.0062470 (PMC3639276; doi:10.1371/journal.pone.0062470)

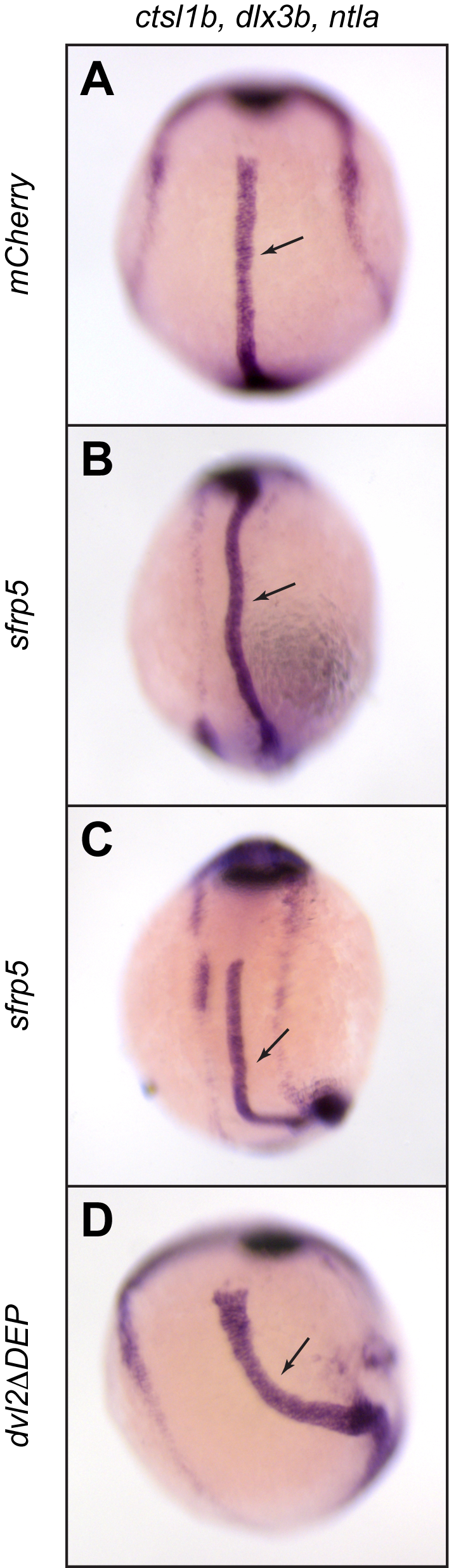

Supplement: Figure S1 — The notochord undulates and is kinked in sfrp5 and dvl2 Δ DEP injected embryos. All embryos were processed by in situ hybridization using a cocktail of probes against ctsl1b, dlx3b, and ntla and are shown in dorsal view, anterior to top. A) Embryo injected with 200 pg of mCherry mRNA. B) Embryo injected with 50 pg sfrp5 mRNA. C) Embryo injected with 140 pg sfrp5 mRNA. D) Embryo injected with 150 pg dvl2ΔDEP mRNA. Arrows point to the notochord. (TIF) [file pone.0062470.s001.tif]

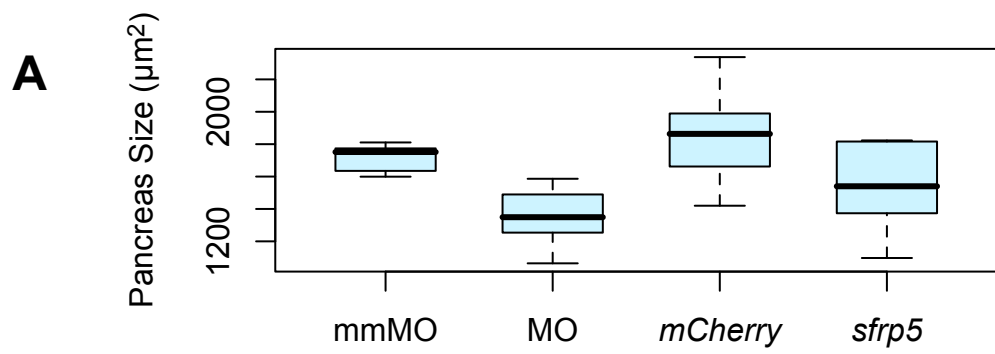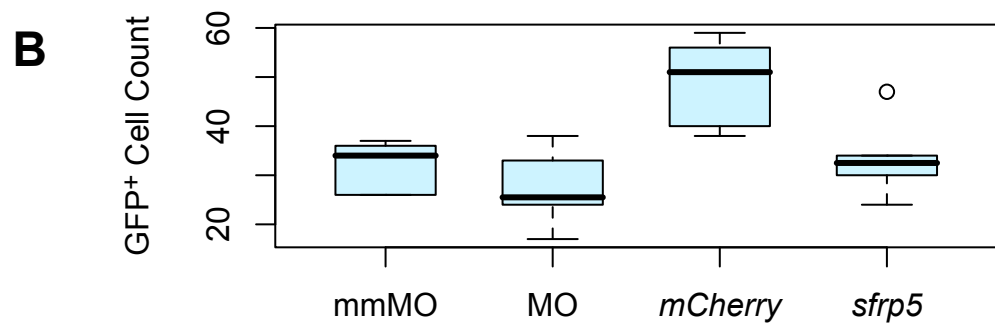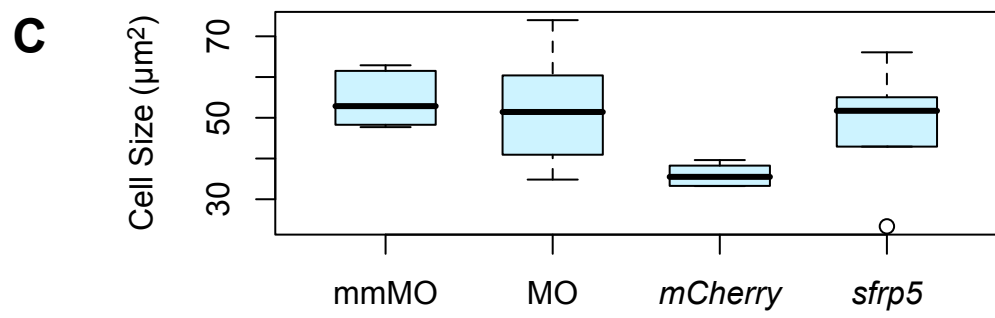

Supplement: Figure S2 — Boxplots showing pancreas size distribution in embryos injected as in Figure 6 . A) Pancreas size in µm2. B) GFP+ cell number. C) Cell size in µm2. The total number of analyzed embryos per treatment is shown below each column. (PDF) [file pone.0062470.s002.pdf]
