# Supplementary material for: Sfrp5 Modulates Both Wnt and BMP Signaling and Regulates Gastrointestinal Organogensis in the Zebrafish, Danio rerio
Source: PLoS One. 2013 Apr 29;8(4):e62470. doi: 10.1371/journal.pone.0062470 (PMC3639276; doi:10.1371/journal.pone.0062470)
Supplement: Table S1 — Primers used in cloning of injection vectors. The table shows the forward and reverse primers used in the cloning of the sfrp5 and dvl2ΔDEP injection vectors along with the region amplified, the GenBank accession number, and ZFIN ID for the respective genes. (PDF) [file pone.0062470.s003.pdf]

Supplemental Table S1: Primers used in cloning of injection vectors.

Protein	Accession Number	ZFIN_ID	Protein Region Amplified (aas)	Forward Primer	Reverse Primer
sfrp5	NM_131858 NP_571933	ZDB-GENE-011108-2	1 – 310 (full length)	GAAAATTTTGATTTACACTTACCC	TCGGAATTAGGCATGGATACAG
dvl2	NM_212648	ZDB-GENE-041118-20	1 – 425 (N-terminal)	CAAGAATTCCAGAACAACGGGAAAGTTTAG	TTCACACATGTCTGAGCGAAGAGACAG
dvl2	NM_212648	ZDB-GENE-041118-20	495 – 747 (C-terminal)	GACATGTGTGAAAACTACATGGCCAACCT	AAAGGCCTTCATTACATCACATCCACAAA
