# Supplementary material for: Sfrp5 Modulates Both Wnt and BMP Signaling and Regulates Gastrointestinal Organogensis in the Zebrafish, Danio rerio
Source: PLoS One. 2013 Apr 29;8(4):e62470. doi: 10.1371/journal.pone.0062470 (PMC3639276; doi:10.1371/journal.pone.0062470)
Supplement: Table S2 — Genes tested by in situ hybridization and primers used. This table shows the genes with their respective GenBank accession number and ZFIN ID that were used as probes for in situ hybridization. For probes that we generated for this manuscript, we also include the forward and reverse primers used. (PDF) [file pone.0062470.s004.pdf]

Supplemental Table S2: Genes tested by *in situ* hybridization and primers used.

Gene	Accession	ZFIN_ID	Forward Primer	Reverse Primer
sfrp5	NP_571933	ZDB-GENE-011108-2	GAAAATTTTGATTTACACTTACCC	TCGGAATTAGGCATGGATACAG
eve1	NP_571189	ZDB-GENE-980526-69		
gsc	NP_571092	ZDB-GENE-980528-2060	GTTGGCAACAAATCTCACACA	GATTCCTCTGACGACGACCTT
chd	NP_571048	ZDB-GENE-990415-33	GGTGTGATGCACTGCGTTATG	GTGAGGTTTCGGCACATTCTT
ctsl1b	NP_571273	ZDB-GENE-980526-285	AGCCAGCATGGAAAAAGCTA	CAGCAGCAACAGCATTCATT
dlx3b	NP_571397	ZDB-GENE-980526-280	ATGAGTTGCCATCCGACTTC	GGTTGACCTGGCTAGAGTGC
ntla	NP_571237	ZDB-GENE-980526-437	CAGAAGGGCAGCGAGAAAGGG	TACGAACCCGAGGAGTGAACA
egr2b	NP_571072	ZDB-GENE-980526-283		
myoD1	NP_571337	ZDB-GENE-980526-561		
her5	NP_571152	ZDB-GENE-990415-90	GGAGGAGAGATCGCATTA	ACAAGCCAAAGCACTATACAG
foxA1	NP_571359	ZDB-GENE-990415-78		
hhex	NP_571009	ZDB-GENE-980526-299	CGCACCCGACGCCCTTCTATATTG	AGTCAGTGGTCAGGTATGGCGGAATG
fabp10a	NP_694492	ZDB-GENE-020318-1	TCAGCGGGACGTGGCAGGTTTAC	GTGGTTCCTCCGACTGTCAGCGTCT
anxa2b	NP_001099070	ZDB-GENE-030131-1089	AATGGCCTTAGTGTCTGAGTATCTG	TGGGAAGGTCTTCACTGCTAGA
ins	NP_571131	ZDB-GENE-980526-110	ATATCCACCATTCCTCGCCTCTGCTT	GGTGGGCAAATCTCTTCAGTTACAGT
try	NP_571783	ZDB-GENE-010131-7	TGTCTCTGAACAGCGGCTAC	AAGTGCAGACCTTGGCGTAAA
