# Supplementary material for: Sfrp5 Modulates Both Wnt and BMP Signaling and Regulates Gastrointestinal Organogensis in the Zebrafish, Danio rerio
Source: PLoS One. 2013 Apr 29;8(4):e62470. doi: 10.1371/journal.pone.0062470 (PMC3639276; doi:10.1371/journal.pone.0062470)
Supplement: Table S3 — Primers used to create the transgenic injection construct and expression vectors for 293T transfection. This table shows the GenBank accession number and ZFIN ID number of genes used in the creation of the injection vector for the transgenic fish line Tg(hs:mCherry,wnt2bb) and for the vectors used in transfecting 293T cells. It also shows the forward and reverse primer used and the region amplified by the primers. (PDF) [file pone.0062470.s005.pdf]

Supplemental Table S3: Primers used to create transgenic injection construct and expression vectors for 293T transfection

Protein	Accession Number	ZFIN_ID	Protein Region Amplified (aas)	Forward Primer	Reverse Primer
wnt2bb	BC162931	ZDB-GENE-060824-6	2 – 396	TGACGTGGAGGAGAATCCCGGCCCTTTCGGCTGCGGTGAAGTT	GGGGACCACTTTGTACAAGAAAGCTGGGTTCAGGTCTGGTCCAGCCATTC
mCherry	ACQ43947		1 – 236	GGGGACAAGTTTGTACAAAAAAGCAGGCTGCCACCATGGCCATCATCAAGGAGTTC	AGGGCCGGGATTCTCCTCCACGTCACCGCATGTTAGAA
T2A				GAGGGCAGAGGAAGTCTTCTAACATGCGGTGACGTGGAGGAGAATCCCGGCCCT	
Insulin	BC005255		1 – 27	CATCAGAAGAGGCCATCAAGCAG	TGTTAACAAAGGCTGCGGCTGGGTC
3x FLAG				CTTTGTTAACACTAGTGACTACAAAGACCATGACGGTGATTATAAAGATCATGAC	TCTAGAGCTAGCACTAGTCTTGTCATCGTCATCCTTGTAGTCGATGTCATGATCT
eGFP	U55761		1 – 815	CCTTTGTTAACACTAGTATGGTGAGCAAGGG	TCTAGAGCTAGCACTAGTGTACAGCTCGTCCATG
Tll1	NP_571085	ZDB-GENE-041020-1	35 – 1022	GTGCTAGCTCTAGAGACTACGATGACAGTTATG	TTACTTCCGTGTGTGTAGCG
Sfrp2	NP_00107085	ZDB-GENE-061013-293	27 – 294	CCTAGCACTGAAGTTTGCGAATG	ACTAGTGCTAGCTCTAGATGGCAGTCGGACAATTTC
Sfrp5	NP_571933	ZDB-GENE-011108-2	36 – 310	ACTAGTGCTAGCTCTAGATGGCAGTCGGACAATTTC	TCACTGGAAGACACTGTGATAGG
